# Correlation of Local FOXP3-Expressing T Cells and Th1-Th2 Balance in Perennial Allergic Nasal Mucosa

**DOI:** 10.1155/2011/259867

**Published:** 2011-11-17

**Authors:** Hideaki Shirasaki, Etsuko Kanaizumi, Nobuhiko Seki, Tetsuo Himi

**Affiliations:** Department of Otolaryngology, School of Medicine, Sapporo Medical University, S-1 W-16, Chu-ku, Sapporo 060-8543, Japan

## Abstract

Regulatory T cells (Treg) play some important roles in allergic rhinitis. The most specific marker for Treg is FOXP3, a recently identified transcription factor that is essential for Treg development. In order to clarify the levels of Treg in allergic nasal mucosa, we studied the relationship between FOXP3-expressing cells and Th1-Th2 balance in nasal mucosa by means of immunohistochemistry. Human turbinates were obtained after turbinectomy from 26 patients (14 patients with perennial allergic rhinitis and 12 patients with nonallergic rhinitis). To identify the cells expressing the FOXP3 protein, double immunostaining was performed by using anti-FOXP3 antibody and anti-CD3 antibody. There was no significant difference in the percentage of FOXP3+CD3+ cells among CD3+ cells in the nasal mucosa of two groups. The proportion of FOXP3+CD3+ cells tend to be correlated positively with GATA3+CD3+ cells/T-bet+CD3+ cells ratio (*R* = 0.56, *P* = 0.04). A positive correlation with GATA3+CD3+/T-bet+CD3+ ratio and FOXP3+CD3+/CD3+ ratio suggests the role of local regulatory T cells as a minimal control of the chronic allergen exposure in nasal mucosa.

## 1. Introduction

The allergic response is a complex process characterized by an aberrant immune response to inhaled environmental allergens. CD4+ lymphocytes with a Th2 phenotype may play some important roles in the development of allergic rhinitis, and the suppression of Th2 lymphocytes could have the potential to be new therapeutic targets for the treatment of allergic rhinitis. The CD4+ CD25+ regulatory T (Treg) cells have been shown to suppress both Th1 and Th2 responses in vitro [1–3]. The most specific marker for Treg cells is FOXP3 as identified transcription factor that is essential for the development of Treg cells.

 Impaired skin infiltration of CD4+CD25+FOXP3+ T cells was observed in acute atopic dermatitis lesions [4], suggesting a dysregulated control of inflammation by Treg cells. With regard to the local FOXP3-expressing cells in allergic nasal mucosa, conflicting results have been reported regarding difference in the levels of FOXP3-expressing cells in between allergic and nonallergic nasal mucosa. In the present study, we evaluated the number of FOXP3+ cells and the frequencies of FOXP3+ T lymphocytes in perennial allergic and nonallergic nasal mucosa by dual-immunofluorescence methods.

## 2. Material and Methods

### 2.1. Subjects

Human inferior turbinates were obtained after turbinectomy from 26 patients with nasal obstruction refractory to medical therapy. Informed consent was obtained from all patients and this study was approved by the ethics committee of Sapporo Medical University. All were nonsmokers, and 14 patients had perennial allergy against mites as defined by questionnaire and CAP test (Pharmacia, Uppsala, Sweden). ENT doctors of our hospital reviewed the questionnaires and determined the clinical diagnosis. Subject without allergies (nonallergic rhinitis: control group) had to satisfy the following criteria: (1) no history of allergic diseases, (2) no detectable, specific IgE antibodies against 4 major inhalant allergens (RAST score < class 1), and (3) total serum IgE levels below the general population mean. All medications, including glucocoriticoids, were prohibited for at least 3 weeks prior to the study. Demographic and clinical characteristics of the patients are summarized in Table 1. The nasal mucosal specimens were fixed in 10% formalin for immunohistochemistry.

### 2.2. Immunohistochemistry

#### 2.2.1. Antibodies

For immunohistochemistry of FOXP3, rabbit anti-human FOXP3 polyclonal antibody (catalog # 623802, BioLegend, CO, USA) was used at 1 : 40 dilutions. For immunohistochemistry of T-bet, rabbit anti-human T-bet polyclonal antibody (catalog#sc-21003, Santa Cruz Biotechnology Inc. CA, USA) was used at 1 : 100 dilutions. For immunohistochemistry of GATA3, goat anti-human GATA-3 polyclonal antibody (catalog#sc-22206, Santa Cruz Biotechnology Inc.) was used at 1 : 40 dilutions. To identify the T lymphocytes, anti-CD3 monoclonal antibody (F7.2.38 clone, Dako Corporation, Carpinteria, CA, USA) was used.

#### 2.2.2. Immunohistochemistry

For double staining of CD3 and FOXP3 or T-bet, deparaffinized sections were stained by immunofluorescence technique. After microwave treatment (10 minutes at 500 Watt in citrate buffer), the deparaffinized sections were incubated in blocking solution (10% normal goat serum in PBS) for 30 minutes before incubation in primary antibody. Then, the sections were incubated with anti-CD3 monoclonal antibody and anti-FOXP3 polyclonal antibody or anti- T-bet polyclonal antibody for overnight at 4°C, washed in PBS, and were incubated for 30 minutes with Alexa Fluor 594-labelled goat anti-mouse IgG (diluted 1 : 50; Molecular Probes, OR, USA) and Alexa Fluor 488-labelled goat anti-rabbit IgG (diluted 1 : 50; Molecular Probes).

For double staining of CD3 and GATA-3, deparaffinized sections were stained by the similar immunofluorescence technique with minor modification. After microwave treatment (10 minutes at 500 Watt in citrate buffer), the deparaffinized sections were incubated in blocking solution (10% normal rabbit serum in PBS) for 30 minutes before incubation in primary antibody. Then, the sections were incubated with anti-CD3 monoclonal antibody and anti-GATA3 polyclonal antibody for overnight at 4°C. After washing with PBS, the sections were incubated with Alexa Flour 594-labelled rabbit anti-mouse IgG (diluted 150; Molecular Probes) and Alexa Flour 488-labelled rabbit anti-goat IgG (diluted 1 : 50; Molecular Probes). After washing with PBS, the sections were mounted with Vectashield mounting medium with 4′,6 diamidino-2-phenylindole (DAPI) (Vector Laboratories Inc, Burlingame, CA, USA) and examined under Olympus BX51 microscope, DP70 CCD camera (Olympus Optical Co., Tokyo, Japan). All images were processed with DP Controller and DP Manager Software (Olympus Optical Co) for image analysis. Negative controls were obtained by replacing primary antibodies by mouse IgG1 and rabbit immunoglobulin fraction (Dako).

#### 2.2.3. Quantitation

For double immunofluorescence staining, the slides were counted using a microscope equipped with an eyepiece graticule. A total of 6 fields (1 mm^2^ each) from each slide were counted by placing the upper edge of the grid at the epithelium. The number of FOXP3+CD3+ cells, T-bet+CD3+ cells, or GATA-3+CD3+ cells in CD3+ cells in allergic and nonallergic nasal mucosa was evaluated by 2 blinded investigators. Results are expressed as the number of FOXP3-positive cells per square millimeter (mm^2^) and the percentage of FOXP3+CD3+cells, T-bet+CD3+cells, or GATA-3+CD3+ cells in CD3+ cells.

#### 2.2.4. Statistical Methods

Values are expressed as mean ±SD. Differences between groups are compared using the Mann Whitney *U*-test. A *P* < 0.05 was considered significant. Linear regression analysis was used to determine the relationship between the percentage of FOXP3+ cells in CD3+ cells and the ratio of GATA3+ CD3+ cells/T-bet+ CD3+ cells in allergic nasal mucosa.

## 3. Results

**Figure 1:**
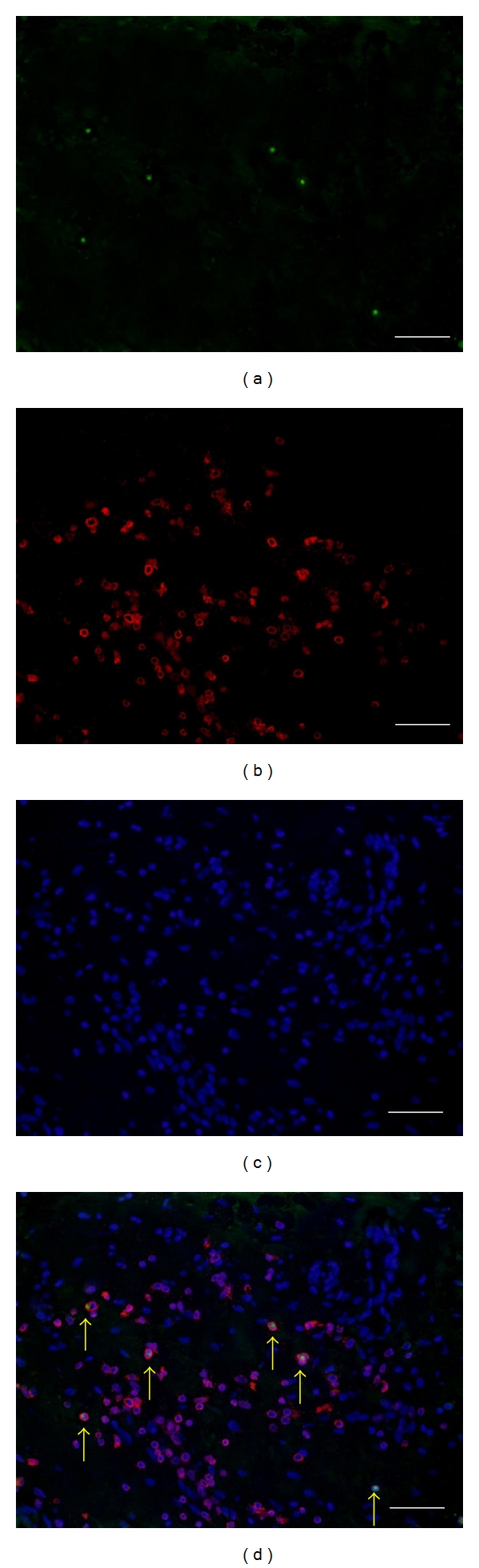
Identification of the cells expressing FOXP3 among CD3 positive cells in human allergic nasal mucosa by means of immunofluorescence technique as follows: (a) the FOXP3 protein (green), (b) CD3 (red), (c) nuclear stain with 4′,6 diamidino-2-phenylindole (DAPI: blue), and (d) The overlay images. The immunoreactivity for FOXP3 was significantly detected in nucleus of some CD3 positive cells. Scale bar = 100 *μ*m.

**Figure 2:**
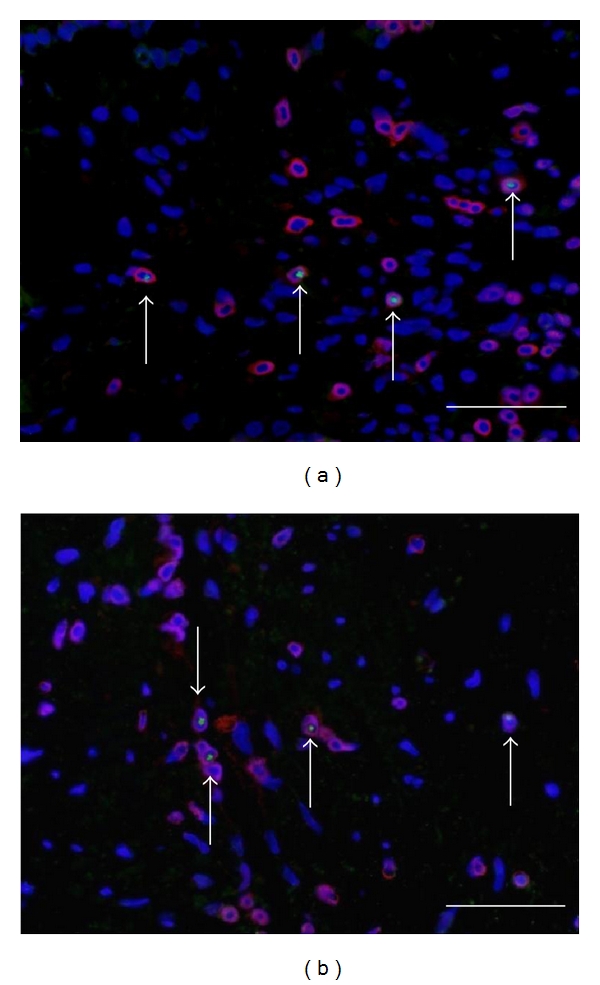
Identification of the cells expressing T-bet and GATA-3 protein in human nasal mucosa. (a) The dual staining for CD3 (red) and T-bet (green) in allergic nasal mucosa. The arrows indicate the cell expressing T-bet in nucleus of CD3 positive cells. (b) The dual staining for CD3 (red) and GATA-3 (green) in allergic nasal mucosa. The arrows indicate the cell expressing GATA-3 in nucleus of CD3 positive cells. Scale bar = 100 *μ*m.

As shown in Figure 1, immunoreactivity for FOXP3 was significantly detected in nucleus of some CD3+ cells. Similarly, immunoreactivity for T-bet (Figure 2(a)) and GATA3 (Figure 2(b)) was significantly detected in some of CD3+ cells in both allergic and nonallergic nasal mucosa. Specificity of the staining was also confirmed by the absence of labeling with normal mouse IgG1 and rabbit immunoglobulin (data not shown).

**Figure 3:**
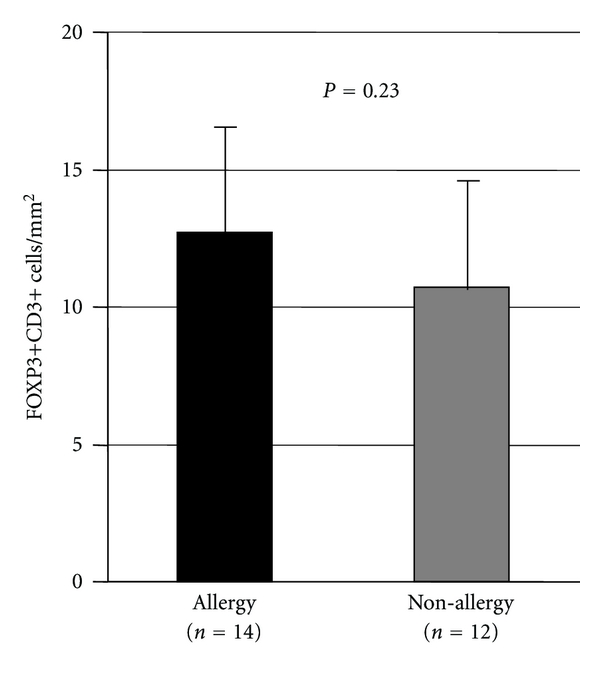
The number of FOXP3+CD3+ cells in allergic and nonallergic nasal mucosa by means of double immunofluorescence staining for FOXP3 protein and CD3. There are no significantly differences between the groups in terms of the number of FOXP3+CD3+ cells.

As shown in Figure 3, no significant difference between the group was found in terms of the number of FOXP3+CD3+ cells (allergic rhinitis: 12.5 ± 4.4, nonallergic rhinitis 10.6 ± 4.0, *P* = 0.23).

**Figure 4:**
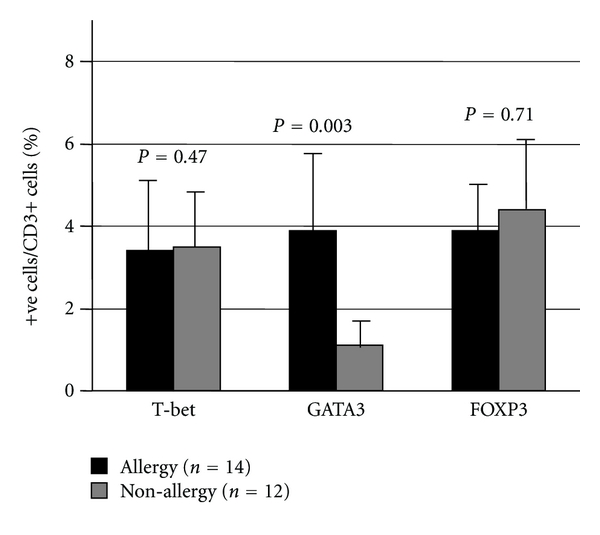
The percentage of T-bet-, GATA3-, or FOXP3-positive T lymphocytes in allergic (*n* = 14) and nonallergic (*n* = 12) nasal mucosa. Sections were stained by double immunohistochemistry (IHC) using anti-CD3 antibody and antibody against each transcription factor protein. The percentage of double-positive cells to IHC-positive cells was calculated. Data are the mean ± SD.

 Next, we compared percentage of T-bet+ CD3+ cells/CD3+ cells, GATA3+CD3+ cells/CD3+ cells, and FOXP3+CD3+ cells/CD3+ cells in between allergic and nonallergic nasal mucosa. As shown in Figure 4, there are no significantly differences in the percentage of T-bet+ T cells and FOXP3+ T cells among two groups. However, the percentage of GATA3+ T cells are significantly higher in allergic nasal mucosa than those in nonallergic nasal mucosa (allergic rhinitis: 3.9 ± 1.8%, nonallergic rhinitis 1.1 ± 0.6%, *P* = 0.003).

**Figure 5:**
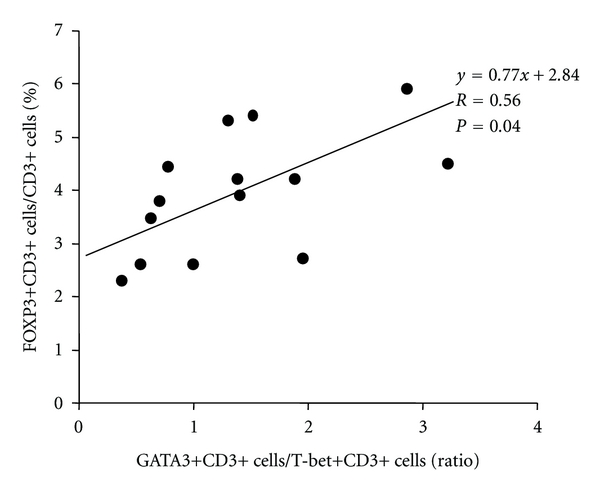
The correlation between the percentage of FOXP3-positive T lymphocytes and Th1/Th2 ratio in allergic nasal mucosa (*n* = 14). Linear regression analysis was used to determine the relationship between the percentage of FOXP3-positive cell in CD3-positive cells and the ratio of GATA-3+CD3+ cells/T-bet+ CD3 cells in allergic nasal mucosa.

Finally, we compared FOXP3+CD3+ cells to Th2/Th1 ratio in allergic nasal mucosa. As shown in Figure 5, the proportion of FOXP3+CD3+ cells in allergic nasal mucosa tend to be correlated positively with GATA3+/T-bet+ cells ratio (*R* = 0.56, *P* = 0.04). The slope of the regression line for percentage of FOXP3-positive T cells against Th2/Th1 ratio was 0.77.

## 4. Discussion

In this study, the average number of FOXP3+ cells in allergic mucosa is higher than that in nonallergic mucosa. However, the difference between the groups is not statistically significant due to high standard deviation in both groups (allergic rhinitis: 12.5 ± 4.4, nonallergic rhinitis 10.6 ± 4.0, *P* = 0.23). With respect to the number of FOXP3+ T cells in nasal mucosa, conflicting results have been reported regarding the difference between in allergic and in nonallergic nasal mucosa. Malmhäll et al. demonstrated that the number of FOXP3+ cells in the nasal mucosa of patients with allergic rhinitis was significantly increased compared with healthy subjects, and that seasonal increases in FOXP3+ cells in nasal biopsy specimens compared with baseline values were observed in both placebo- and immunotherapy-treated patients with grass pollen allergy [3]. In addition, nasal challenge with pollen extract increased the number of FOXP3+ T cells at the inflammatory site in pollen-allergic patients [5]. These reports suggest that local allergen exposure induces the increases of local FOXP3+ T cells [6].

On the contrary, another study has demonstrated that nasal secretions of patients with allergic rhinitis had significantly lower FOXP3 mRNA compared to nonallergic controls [2]. From these observations, further studies will be necessary to determine whether the upregulation or downregulation of local T-regulatory cells may exist in patients with allergic rhinitis.

In the present study, we focused on the local Th1/Th2 balance and regulatory T cells, and our study demonstrates a positive correlation between FOXP3+CD3+ cells/CD3+ cells and GATA3+ CD3+ cells/T-bet+ CD3+ cells ratio. It has been well known that IL-4 is one of the most important cytokine on the local Th1/Th2 balance, because IL-4-induced GATA3 is associated with Th2-type responses and IL-4 has already been shown to negatively regulate the development of naïve T cells into Th1 or the IL-17-producing T cells (Th17)[7]. It is well known that FOXP3+ T cells accumulate in a variety of human inflammatory disorders. One possibility, of course, is that the accumulation of FOXP3+ cells, at least in part, depends on local proliferation induced by TGF-*β* and other cytokines. With respect to the effect of IL-4 on the induction of FOXP3, conflicting results have been reported in mice in vitro. Skapenko et al. demonstrated that IL-4 induced the generation of FOXP3+ regulatory T cells [8]. This observation may support our present data. On the other hand, Mantel et al. reported that FOXP3 expression was negatively regulated by IL-4 [9]. Whether or not Th2 cytokines such as IL-4 directly affect the accumulation of the regulatory T cells is still unclear and needs to be clarified.

 There have been several reports regarding the circulating regulatory T cells by mean of flow cytometory. It was found in peripheral blood of pollen-sensitized children that Treg cells increase during the pollen season [10]. Circulating Der p1-specific CD4+CD25^hi^FOXP3+ Treg cells obtained from atopic patient inhibited antigen-induced proliferation of T cells [11]. These results suggest that the Treg cells keep the inflammation at low levels. Another study found that circulating FOXP3+/CD4+ ratio was significantly low in patients with asthma and atopic dermatitis [12]. Whether these Treg cells directly contribute to allergic inflammation or not remains an important research question.

Allergen-specific immunotherapy, which consists of repeated subcutaneous or sublingual administration of allergen, has been widely used and proved effective for the management of allergic rhinitis. The mechanism of the therapeutic effect of allergen-specific immunotherapy is not completely understood but might involve the activation and expansion of Treg cells. It is well established that FOXP3 acts as master transcription factor for naturally occurring regulatory T cells (nTreg) development and function [13].

Several mechanisms involving Treg in tolerance induction after allergen-specific immunotherapy have been documented. Such mechanisms include increased capacity of Treg to suppress Th1 and Th2 cells [14], induction of IL-10 and TGF-beta [14], decreased allergen-stimulated T cell proliferation [15], or suppression of effector cells [16]. The induction of a tolerant state in peripheral T cells represents an essential step in allergen-specific immunotherapy. Peripheral T cell tolerance is characterized mainly by generation of allergen-specific Treg cells and suppressed proliferative and cytokine responses against the major allergen. It is initiated by the autocrine action of IL-10 and TGF-*β*, which are increasingly produced by the antigen-specific T_R_1 cells [17]. It was shown that grass pollen immunotherapy increased the expression of mucosal and peripheral T-cell IL-10 [18] and TGF-*β* [19]. It has been shown that the increased number of FOXP3+CD25+ Treg in nasal mucosa after grass pollen immunotherapy correlated with clinical efficacy and suppression of seasonal allergic inflammation, thus supporting the role of Treg in the induction of allergen-specific tolerance in human subjects [6]. From these reports, we suppose that the exposure of allergen locally or generally may increase the number of Treg cells in nasal mucosa of perennial allergic rhinitis patients. So, further studies on nasal mucosa from the perennial rhinitis patients who received immunotherapy will be necessary to clarify the roles of immunotherapy on the local Treg cells and Th1-Th2 balance in perennial allergic rhinitis.

In conclusion, we have demonstrated a positive correlation with GATA3+CD3+ cells/T-bet+CD3+ cells ratio and FOXP3+CD3+ cells/CD3+ cells ratio, suggesting that Treg cells are operative on Th1-Th2 balance in perennial allergic nasal mucosa. Our present findings should be of considerable interest for understanding the role of regulatory T cells on upper airway diseases such as allergic rhinitis.

## Figures and Tables

**Table 1:** Demographic characteristics of perennial allergic and nonallergic patients.

	Perennial allergic rhinitis	Nonallergic rhinitis
	*n* = 14	*n* = 12
Sex (male/female)	6/8	6/6
Age	38 (19–65)	36 (19–58)
Specific IgE to house dust mite (d1) (kU/L)	3.3 (1.0–26)	<0.35
Specific IgE to birch (t3) (kU/L)	0.15 (0–2.4)	<0.35
Specific IgE to orchardgrass (g3) (kU/L)	0.23 (0–1.1)	<0.35
Specific IgE to mugwort (w6) (kU/L)	0.20 (0–2.1)	<0.35
Total IgE (kU/L)	235 (10–523)	101 (10–210)
Blood eosinophils (cells/*μ*L)	378 (70–740)	110 (45–240)
Current nasal symptoms (number of patients)		
Nasal obstruction	14 (all patients)	12 (all patients)
Sneezing	8	1
Rhinorrhea	6	3

Data expressed as median values and range (in brackets).
